# An Easy to Deploy Street Light Control System Based on Wireless Communication and LED Technology

**DOI:** 10.3390/s130506492

**Published:** 2013-05-16

**Authors:** Pilar Elejoste, Ignacio Angulo, Asier Perallos, Aitor Chertudi, Ignacio Julio García Zuazola, Asier Moreno, Leire Azpilicueta, José Javier Astrain, Francisco Falcone, Jesús Villadangos

**Affiliations:** 1 Deusto Institute of Technology (DeustoTech), University of Deusto, Bilbao 48007, Spain; E-Mails: pilar.elejoste@deusto.es (P.E.); ignacio.angulo@deusto.es (I.A.); achertudi@deusto.es (A.C.); i.j.garcia@deusto.es (I.J.G.Z.); asier.moreno@deusto.es (A.M.); 2 Electrical and Electronic Engineering Department, Universidad Pública de Navarra, Pamplona 31006, Spain; E-Mails: leyre.azpilicueta@unavarra.es (L.A.); francisco.falcone@unavarra.es (F.F.); 3 Mathematics and Computer Engineering Department, Universidad Pública de Navarra, Pamplona 31006, Spain; E-Mails: josej.astrain@unavarra.es (J.J.A.); jesusv@unavarra.es (J.V.)

**Keywords:** street lighting, sensor networks, wireless technologies, intelligent systems, context-aware system, smart city

## Abstract

This paper presents an intelligent streetlight management system based on LED lamps, designed to facilitate its deployment in existing facilities. The proposed approach, which is based on wireless communication technologies, will minimize the cost of investment of traditional wired systems, which always need civil engineering for burying of cable underground and consequently are more expensive than if the connection of the different nodes is made over the air. The deployed solution will be aware of their surrounding's environmental conditions, a fact that will be approached for the system intelligence in order to learn, and later, apply dynamic rules. The knowledge of real time illumination needs, in terms of instant use of the street in which it is installed, will also feed our system, with the objective of providing tangible solutions to reduce energy consumption according to the contextual needs, an exact calculation of energy consumption and reliable mechanisms for preventive maintenance of facilities.

## 1. Introduction

Street lighting in Spain accounts for 10% of the total energy consumption in lighting and stands at 116 kW per year and inhabitant [1], compared with the 91 kW or the 43 kW in France and Germany, respectively [2]. The latest available figures from the Ministry of Industry, Tourism and Trade assume an electric power consumption of 3,630 GWh/year for the whole of Spain. It is worth noting that, in total, there were an estimated 4,800,000 points of light in 2010, and a third of street lighting is based on outdated and inefficient technologies.

Drawing on energy sector studies, public lighting is has the greatest impact on energy consumption of a municipality, and may account for up to 54% of total energy consumption and 61% of electricity consumption of municipal facilities. The importance of public lighting installations is such that, in some municipalities, this item now accounts for up to 80% of electricity consumed and up to 60% of the energy consumption budget of the municipality.

In the particular case of Spain, a legal framework in the form of a Royal Decree 1890/2008 [3] and its corresponding Complementary Technical Instructions was approved on 14 November 2008 by the Ministry of Industry, Tourism and Trade. The main objectives of the Royal Decree are: (1) to improve energy savings and efficiency, and consequently, reduce greenhouse-effect gas emissions, (2) to limit glare and light pollution, because of too much light and/or the use of unsuitable luminaries, and (3) to reduce intrusive or annoying light. This Royal Decree is mandatory and it is part of the Spanish Government's 2008–2011 Energy Saving and Efficiency Plan, a strategy called E4 (2004–2012 Energy Saving and Efficiency Strategy) [4] which establishes a series of standard actions aimed at improving the energy system in Spain. The target set in this Plan was to achieve a consumption of 75 kW per inhabitant per year, a major challenge considering that no Spanish province has currently reached that objective.

Based on the aforementioned considerations, innovation in efficient streetlight management systems is a must. We propose a scalable, holistic and efficient solution that provides lighting only when necessary (according to the instant weather conditions or the presence of persons and vehicles) with the objective of reducing the related cost in the municipalities, helping the economic recovery. The illumination level will be conveniently regulated, thus avoiding overlighting and glare. The installation cost will be reduced to the minimum, using wireless communications and autonomous performance modes. The predictive monitoring of each spotlight would also reduce the price of the streetlight installation maintenance. All these improvements have to be considered in order to achieve a significant reduction of the energy consumption in lighting and consequently contribute to sustainable development.

The rest of the paper is organized as follow: Section 2 introduces existing research efforts and commercial solutions in a brief state of the art summary; Section 3 presents the functional achievements, while Section 4 introduces the system architecture; Section 5 describes the current system implementation; then some preliminary test results are shown in Section 6; this system will be improved with two works in progress advanced in Section 7; and finally, Conclusions, Acknowledgements and References end the paper.

## 2. State of the Art

Research efforts in this area, as indicated in [5], have focused on the use of new technologies for the light sources (such as LED technology) [6–9] and on the development of different remote-control system based on intelligent lamp posts that send information to a central control system in order to simplify management and maintenance issues [10–17]. There exist also several intelligent streetlight energy management solutions such as Smart Street Lighting [18], IllumiWave [19], Street Light Control (SLC) [20] or iiLuix [21], which enable remote control and management of widely distributed streetlights from a central management system. Those research and commercial systems enable to remotely scheduling streetlights to turn off or dim when needed for energy savings purposes. They also provide information concerning energy consumption, alerts for bulb outages or abnormal bulb operation, maintenance and repair orders, and many others. The centralization degree of these proposals concerns many issues as the communication technology used as General Packet Radio Service (GPRS) [22,23] and Power Line Communications (PLC) [24,25], the system architecture proposed (centralized [26], distributed [27,28] or hybrid), and the data sensing and fusion strategy followed.

In addition, these systems often require the installation of new lights [29] hindering their deployment in functional facilities. The overcrowding of multiple sections of lights in the same electric cabinet, coupled with the significant loss of tension on power lines, causes serious interference to PLC based systems [14,30], that sometimes prevents their proper operation in poorly sized installations. Moreover, the evaluated systems [31–33] based on wireless communications, provide general solutions which do not take into account the particularities of the specific scenario where system must be deployed. The specific characteristics of electromagnetic propagation environment must be taken into account in the deployment of each section system in order to solve physical barriers and other issues affecting radio communication.

Our proposal describes an autonomous system, where a holistic and “bottom-up” approach to system design (from physical to application layers) is presented, which ensures standardization, interoperability and adaptation to many needs and scenarios. This is a non-functional but distinguishing aspect compared to other mentioned solutions, which are designed specifically for a concrete scenario, following a “top-down” approach, and with little chance of migration to another environment.

We propose an intelligent street lighting system where, contrary to other proposals described above such as those of [5,16], a lower number of sensors are required to be incorporated in spotlights, where the sensed data aggregation is performed using a mesh strategy instead of a chain strategy [5] in order to maximize fault tolerance and ensure a complete fusion of information, and where nodes can work autonomously since they have enough intelligence to detect communication problems with the remote control system or situations that require an immediate action over the spotlight. Even though remote control system will gather off-line data, receive incidents and provide decision support tools its workload is the least, a relevant difference with other approaches, in which scalability and performance are highly compromised.

In addition, the accurate radio-electrical analysis of global scenarios enables the obtaining of valuable information related to capacity/coverage of wireless communications that eases the design of optimal networks in terms of performance. The open architecture which we have set out will create a robust, secure and flexible networking environment, in order to meet the World's digital demands. The combination of advanced wireless communications, sensing and metering capacities in a unique infrastructure will contribute to the deployment of the future Smart Cities.

## 3. Functional Achievements

The main challenge to be achieved in our work is the development of a system capable of becoming a set of streetlights smart enough to work autonomously, as well as to be able to be remotely managed (for diagnostics, actions and other energy optimization services). These two capabilities can contribute decisively to improve the global efficiency of the lighting systems, both from an energy consumption point of view as in the cost required for their maintenance. This section covers the requirements that have to be fulfilled in order to meet these challenges.

First, the autonomous operation of the streetlights requires that their behavior can be changed according to the environmental conditions. Thus, they have to be able to perceive some relevant characteristics of the environment, such as ambient lighting, presence of vehicles or people, or their own performance (*i.e.*, system diagnosis). Therefore, streetlights have to be equipped with some kind of sensors. Second, streetlights have to be able to act according to the knowledge obtained from the environment. In this case, action is translated into a lighting intensity regulation, being able to perform at a lower level when it is not necessary.

Finally, the smart operation of an individual streetlight also requires some knowledge about what is concurrently happening in the nearest ones, as well as what happened in the past and what the consequences of previous actions under similar conditions were. That is to say, a holistic knowledge which can be only supported by a remote control center to which streetlights report their local knowledge. Thus, streetlights not only need sensors and lighting regulators, but must also communicate with an external management center, a mandatory requirement for the centralized strategy. This last capability is also necessary to carry out the previously mentioned remote management capability.

On the other hand, due to cost considerations, the inclusion of a high number of sensors and a long range communication device (such as a GPRS modem) in each individual streetlight is not a feasible approach. Instead, then use of collaborative techniques could be an intelligent way of reducing the total cost of the system. In that way, only a smaller amount of streetlights would be equipped with sensors and long range communication devices (those which have the best location according to sensorial and communication requirements). The rest would only include a device needed to configure a mesh network between streetlights in the same geographical or electrical region. Their behavior would be coordinated by the smartest street-lights which would gather data from the environment and communicate with the control management on their behalf.

## 4. System Architecture

**Figure 1.**
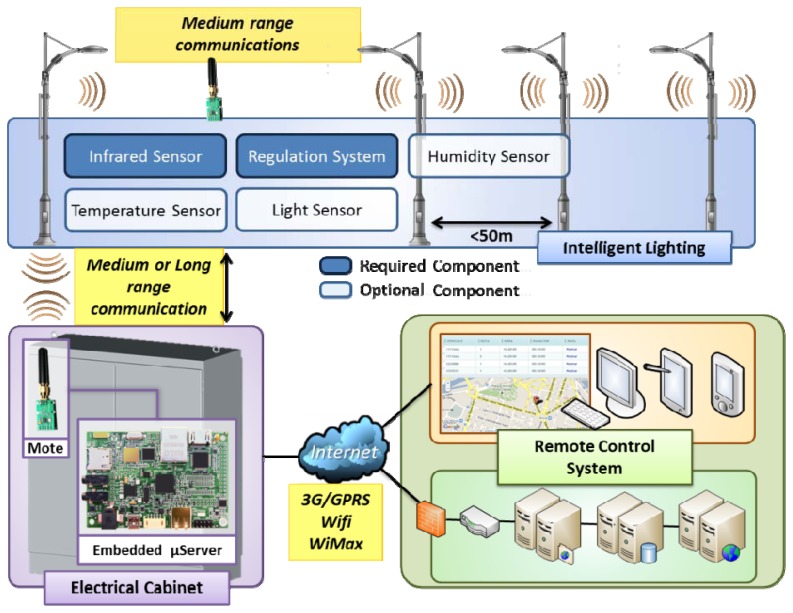
Architecture of the system.

The proposed intelligent streetlight system has been designed following a three-level hierarchical model. This model has been chosen to ease the physical deployment of the solution in existing facilities ensuring system scalability. This approach allows, for example, changing the communication channel between nodes with a minor impact in the rest of the system. Figure 1 illustrates the elements of the system as a whole that will be described in the following paragraphs.

The lower level is composed by motes or end nodes. These devices are integrated on all the lamps in a section providing the computing power needed for its control and regulation [3]. The motes include wireless transceivers to communicate with the other lamps of the section creating a mesh-type network among them. One of the considered key factors lies in using wireless channels in order to perform the transactions of data between nodes. This characteristic is also useful for new or old lighting installations. The civil works needed for powering the luminaries in a section, manholes and connections to the electrical panel usually represent an important amount of economic resources in street lighting installations. At this level the system's sensing capacity is established, allowing the whole network to successfully measure and characterize environmental changes. This capability could be located in all the nodes, but due to cost considerations, the required sensors are only incorporated in a few spotlights of each installation, in order to guarantee the knowledge of the surrounding weather conditions without compromising the real deployment spending.

In some instances, the long distances between electric panels and sections of streetlights can exceed the physical capabilities of most wireless communication protocols. Furthermore, the existence of obstacles (buildings) between the street light installations and their corresponding electrical panels reduces noticeably the reach of long-range communications. For those situations, considered as worse case performance scenarios, the proposed system allows some of the end nodes to be equipped with a second transceiver and communicate with the cabinet *via* long range wireless communication. These special nodes, called “reflectors”, are placed at specific points of the infrastructure. Those nodes act as bridges increasing the coverage of the section network.

The second level of the hierarchy is composed of remote concentrators located in the electric cabinets that power each light section. An embedded micro server system is installed to control all the spotlights integrated on the system that are powered by the cabinet. This is the intermediate level of the hierarchy and provides connectivity to both remote control system and end nodes. For this reason, it is an interesting piece of the system by managing relevant information including:

- Sensor acquisition data, such as instant temperature, humidity, direct lighting, *etc.*
- Electrical measurements taken in the main power entrance, for example, total energy consumption for each street lighting section in consideration, voltage and current measures at important points in the electrical panel.
- Individual data for each spotlight, such as information about individual components or devices of the embedded systems in each luminary: life hours, energy consumption, status for the critical inputs/outputs, battery level is apply, last hour detections.

These data are transferred through mobile broadband network or, if possible, using existing WiFi or wired connections, to the Internet and from there to the remote control system's resources. Of course, when malfunctions (failure to function, abnormal functioning, nodes loss, and so on) occur, they will be notified to the remote server as critical events, aiming to solve the problems as soon as possible. The highest level of the hierarchy consists of the remote control system, whose tasks can vary regarding to three applicable strategies.

In the first place, adopting the traditional solution, where the system control is under one remote supervisor's domain at the municipality's offices, all the system intelligence develops far away the actual street lighting section. The system management is performed by the remote control system, which, employing preconfigured policies or manual control, will arrange the different installations in order to manage of their energy consumption. It will be able to modify the seasonal hours for the “power-on” and “power-off” of the whole section and activate or deactivate the light regulation, setting unalterable maximum and minimum limits of light intensities.

Alternatively to the previous case, if a totally distributed control strategy is adopted, nodes provide responsive sensors independently without requiring supervision of a higher order entity (autonomous response to changing environment and situations).

Finally, a hybrid approach is possible assuming that each lighting section has the capacity to self-configure. The information received by the nodes from concentrator is completed with the data acquired from the environment to meet specific policies established by the control center.

Almost all of the available smart street lighting energy management solutions build a centralized control center that gathers the data obtained from the deployed systems and makes the decisions remotely. This adds another problem, *i.e.*, the need of huge quantities of data received and the need for quick analysis by the remote control system, in order to give an optimal response to the changes detected in every installation. On the other hand, all the intelligence can be allocated at the end nodes, giving them the capacity of taking decisions and managing each spotlight as an independent system, as an innovative and distributed approach. In this work a third approach is introduced, that can minimize the data transaction volume, allowing the end nodes taking some control in the tasks of light management and regulation, in order to reduce the computational requirements of the remote central system, giving more intelligence to the nodes, that are not yet merely sensors or actuators, but also local managers of their system. In order to distinguish it from the previous one, we will consider it as a hybrid strategy.

### 4.1. Sensor Capacity System

To enable proper control of the illumination of the lamps it is necessary to provide some sensing capability to the end nodes in order to obtain environmental information to minimize energy consumption. In the approach followed in this paper, weather conditions which will be taken into account in order to modify the emitted light level and hence help to optimize the system's consumption and performance. Ambient luminosity, instantaneous temperature and humidity levels, accumulated rain quantities and their cross-analysis with other local weather sources can establish special situations when extra illumination is needed, or otherwise, allow the reduction of light levels when outdoor lighting is not really required. Other key environmental factors could be measured as well, if needed in specific locations (pollution level, gases concentration, and so on). Regarding the street lighting section to consider, these sensing capabilities will be deployed in selected spots, balancing between the cost of end nodes and the quality and reliability of environmental measurements.

Another important factor of the system is to detect the flow of pedestrians and vehicles in the surroundings of each section. Depending on the nature of the detection to be performed, the technology to implement varies. For pedestrian detection, and always avoiding any civil work derived of its installation, simple Passive Infrared Sensors (PIRs) in each luminary would be sufficient. In the case of vehicle detection, several solutions could be applied, depending of the topology and characteristics of the concrete lane: radar, vision, *etc.* The intelligent system could also use, if necessary, data collected from electromagnetic loop detectors situated along roads, which detect vehicles when they pass over them and collect the data required for traffic control: counts, queues, saturation, occupancy rates and intervals between vehicles.

### 4.2. Light Regulation

Due to their properties, LED based luminaries are the best solution for indoor and outdoor lighting applications. LEDs, or light-emitting diodes, are a form of solid-state lighting that is extremely efficient and long-lasting. For optimal performance, they must have reliable drivers that match the long lifetime of the LEDs. The designed embedded system must have the minimal required signals to interface with the driver that powers the led bars of each luminary. Most of commercial providers offer current/voltage constant drivers (which provide constant current output, optimal for a single string of LEDs) as well as regulated ones (programmable drivers that deliver some or all dimming options and a range of currents in a single driver) [34]. System reliability is maximized by monitoring the LED bar temperature. For our system's requirements, the last option (the dimming or programmable driver) should be selected, in order to change the driver output at will.

According to the specific luminary, their provider's full portfolio should be analyzed in order to select the appropriate driver, offering maximum flexibility with customizable operating settings, with the aim of achieving the optimal performance tailored for each design.

The key to overall energy savings achievable by the proposed system is to regulate the light intensity of each lamp inside a streetlight section depending on their specific needs. The multiple technologies used previously deployed sections of streetlights prevents the use of a common dimmer, being necessary to custom design the regulation device to cover the needs of the lights installed.

### 4.3. Remote Control System

As it was previously stated, several currently available systems allow the remote management of streetlight facilities. However, issues such as interoperability, scalability or accessibility are key challenges to deal with in order to optimize their performance.

First, it is desirable that the software management solutions can be easily migrated to other use scenarios. Thus, these solutions should be able to deal with the integration of heterogeneous lamp control technologies and middleware and also multiple applications related to a Smart Cities context, such as traffic, weather or motion monitoring, among others. Thus, portability not only has to concern streetlight management but also other domains. Adopting general purpose approaches such as supervisory control and data acquisition (SCADA) systems, but improved through the use of friendlier Rich Internet Application (RIA)-based interface, could be a good approach, due to the fact that these kinds of computer-aided industrial control systems are technically mature and widely adopted.

Second, the system should be capable of scaling to an expandable infrastructure that supports a larger number of lamps. For this purpose, a distributed approach of the system intelligence is the most appropriate option due to the fact that the workload is not concentrated at a unique point, being more difficult to overload.

In this case, the remote control system has to be able to configure the rules which define the behavior of the different lighting sections and send them to the delegated concentrator node in order to apply them in responsive operations. This collaboration between them is a key issue to guarantee an autonomous behavior of the system and its scalability. In that way, this remote control system will only be responsible for periodically gathering off-line data sent by the set of concentrator nodes, receiving incidents and providing the decision support tools needed to analyze the set of data gathered by the deployed sensors. Therefore, its workload is clearly reduced, representing a very scalable architecture because the performance of the system does not depend on the number of geographical lighting sections in which the system is deployed. This is a relevant difference with other approaches, adopted in most of available market products, where the autonomous responsive behavior requires the supervision of a higher order entity. Thus, the scalability and performance of the solutions is highly compromised.

Finally, the way in which the services of the central control system are accessed is also a key point to provide high added value. Enabling information access using mobile devices such as smartphones or tablets for maintenance task support or the adoption of web-based user interfaces to provide seamless access to the system, are useful benefits. Moreover, through its control panel the system should be easy to configure and operate. Adoption of a Geographical Information System (GIS)-based approach, enabling one to easily map geographical areas to lighting sections, to configure their behavior and access related information, provides a good user-centered design.

## 5. System Implementation

A specific street light control system based on the design presented in Section 4 has been implemented according to the requirements of a particular scenario. In this section the implementation of the hybrid approach of such system, the test scenario where it has been validated, as well as the test results are described.

### 5.1. Topological Characteristics of the Test Scenario

The selected street section has an estimated length of 200 m and is located on Areta Street, a stretch of road connecting the city center of Llodio with the district of Areta in Spain. It is a recently renovated area with LED lighting that allows adjustments without changing the technology of existing facilities. In the electrical panel board, its power supply is separated from the rest, so accessibility and the management of this installation is easier than in shared facilities. The roadway has two lanes for moving traffic and provides for two-way movement of traffic. At the beginning of the section there are a traffic light and a crosswalk. All along the road, the sidewalk where the streetlights which are going to be regulated are located is on only one side.

**Figure 2.**
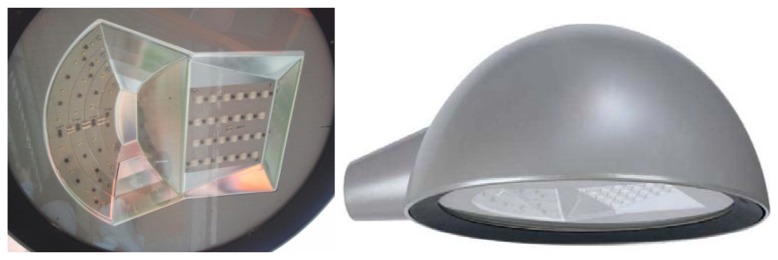
Picture of the streetlights on real scenario.

The tested facility has nine points of light separated by 20 m from one to the next (manufacturer SIMON Lighting, model ALYA LED, which main characteristics are shown in Table 1 and Figure 2), ideal for urban lighting applications [35]. For more details, see below the aerial photography of the selected scenario (Figure 8).

The distance to the electrical panel (represented by spot A in Figure 8) is about 300 m from the nearest luminary (spot B in Figure 8), located at the rear of a residential area. Therefore, it entails adverse conditions for the establishment of a communication link and its integrity preservation (private access points, distance, and interference).

### 5.2. Radio-Electrical Analysis

**Figure 3.**
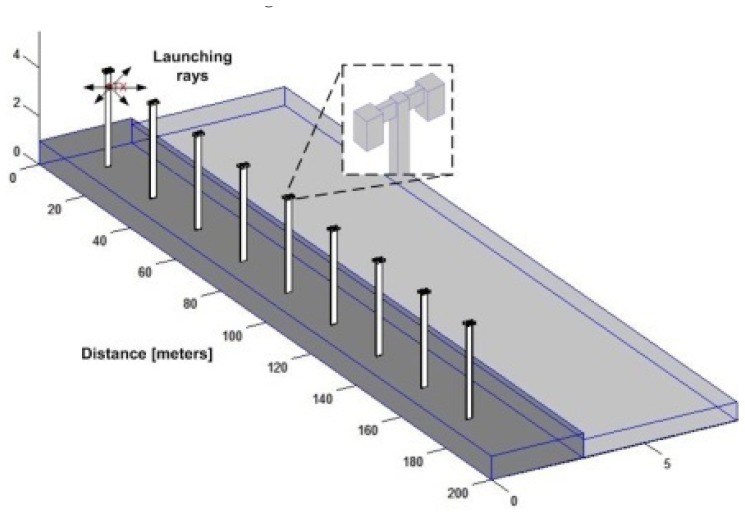
Schematic scenario.

A deterministic method based on 3D ray launching is used to analyze the behavior of the wireless system of the considered scenario. The assessment of the prevailing electromagnetic spectrum is of importance in order to model overall performance of the system under analysis in terms of coverage and capacity analysis. The real and schematic scenario are represented in Figure 3, which consists on a stretch of road 200 meters long with nine streetlights evenly distributed along the street on one side of the road.

The use of deterministic modeling leads to an optimal sensor configuration to provide a competitive, flexible and scalable solution. Simulations have been done with the aid of a 3D ray launching algorithm [36] implemented within our research team, based on Matlab™ programming environment. Several transmitters can be placed within a scenario, in which power is modeled as a finite number of rays launched within a solid angle. Parameters such as frequency of operation, radiation patterns of the antennas, number of multipath reflections, separation angle between rays and cuboids dimension are introduced. Phenomena such as reflection and refraction are considered based on Geometrical Optics (GO) theory. The diffracted rays are introduced with the Geometrical Theory of Diffraction (GTD) and its uniform extension, the Uniform GTD (UTD). The purpose of these rays is to remove the field discontinuities and to introduce proper field corrections, especially in the zero-field regions predicted by GO. The material properties for all the elements within the scenario are also considered, given the dielectric constant and the loss tangent at the frequency range of operation of the system under analysis. This simulation technique is optimal in terms of precision and required calculation time, due to the deterministic nature of the simulation and the geometrical optics approximation [37]. Table 2 lists the parameters used in the simulation.

**Figure 4.**
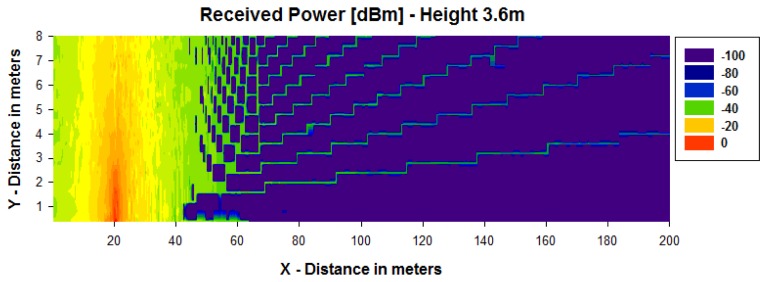
Estimation of received power (dBm) for a height of 3.6 m.

**Figure 5.**
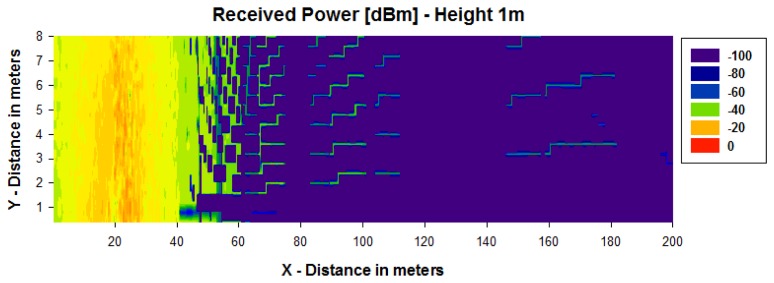
Estimation of received power [dBm] for a height of 1 m.

Figures 4 and 5 show simulation results obtained for the received power for different heights using a transmitter antenna from Libelium (802.15.4 PRO 5dBi) placed in the first streetlight at a height of 3.6 m (see Figure 3) by means of an in-house 3D ray launching algorithm [38]. It is observed that the received power is higher for the same height as the transmitter antenna (Figures 4 and 5), and the transmission power decreases with distance with great variability.

**Figure 6.**
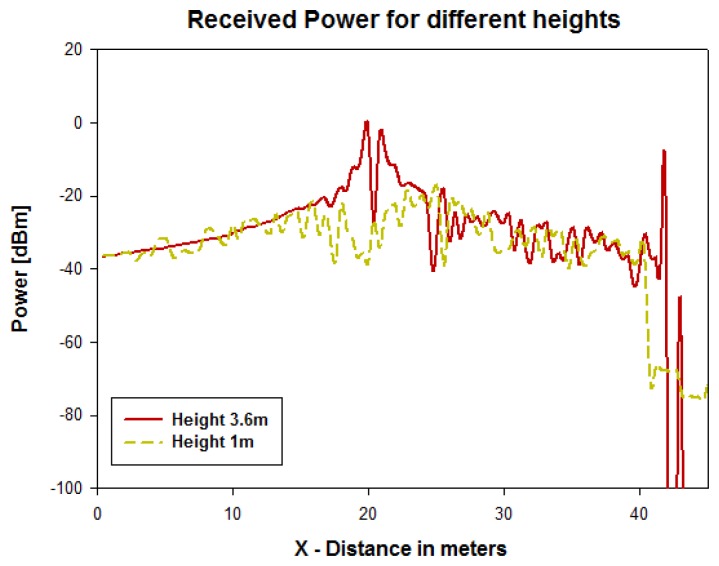
Radials of received power for different heights.

**Figure 7.**
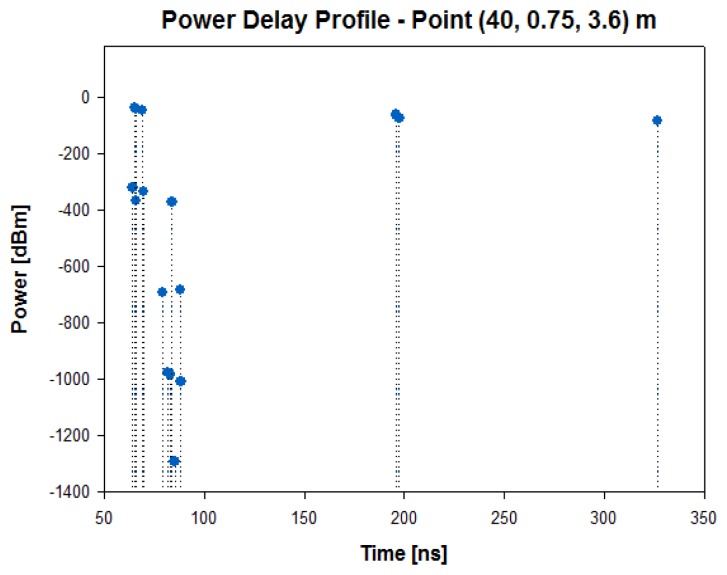
Power Delay Profile at the second streetlight for a height of 3.6 m.

In the second streetlight, which is placed at a distance of 40 meters, the received power level for a height of 3.6 m is around −40 dBm, as it is observed in Figure 6, which represents the radials of received power for both heights through 50 meters of distance. To illustrate the relevance in this propagation channel, the power delay profile at the second streetlight for the same height of the transmitter antenna is depicted in Figure 7. A large number of echoes in the scenario in a time span of approximately 50 to 350 ns may be observed.

### 5.3. Network Topology

The implemented network topology is composed of several nodes, each one of them formed by a luminary and the mote that controls it. The Libelium motes include XBee 802.15.4 transponders with DigiMeshTM firmware [39] for the 2.4 GHz band, the option selected in order to establish mesh networks. The aim of the DigiMeshTM proprietary firmware was to fit together several features (self-healing, route discovery, use of selective ACKs, the sleep modes and multi-hop connections), in an easy to use peer-to-peer protocol, which adds support for dense networks and sleeping nodes and optimizes the definition of the network. Every node is identified with a unique number associated to the XBee transponder (in fact, the MAC address) and a *NodeID* (node identifier) given for this application. The *NodeID* has been set up to a two bytes length attribute (or four hexadecimal characters) related to the section and the particular luminary where the node is located.

All of the nodes of a streetlight line, along with the remote concentrator that controls them (*i.e.*, the intermediate level of our hierarchy), form a mesh network that enables the communication between them. Each mesh network has assigned a PAN ID (two byte length, up to 65,534 possible networks, considering large systems), in order to prevent one section messages from interfering in another. This assignment will be automatically performed when a new line is created, but some identifiers are reserved (especial PAN IDs are defined for the inclusion of new nodes in an existing system).

One remote concentrator can control all the streetlights located in certain blocks, managing their different networks (PAN IDs) and storing all the information collected by the nodes. Besides, it communicates via TCP/IP with the control system server, the third element in our hierarchy. This communication serves a dual purpose, to dump all the data collected by the concentrators to the remote system, but also to allow the control server to access and remotely control both complete facilities as well as its individual elements. In this way, the proposed system follows a hybrid performance mode, halfway between the centralized and the autonomous mode.

**Figure 8.**
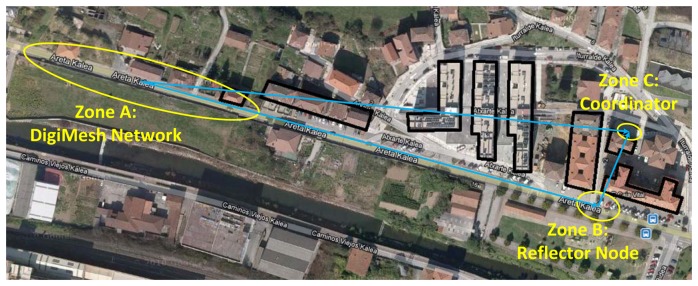
Location of the nodes and concentrator on the selected scenario.

In this particular scenario, the large distance existing between the installation (located in Zone A, Figure 8) and the Control Panel (located in Zone C, Figure 8), as well as the several buildings between them (marked with black outlines in the same picture), prevents this direct communication and compromises the application functionality. This problem has been solved by adding an extra node in Zone B, which works as a repeater allowing the communication between the two distant nodes. This node will exclusively have gateway functions, without having any sensor connected.

The system performance takes into account special situations, such as the addition or replacement of nodes with new ones, for maintenance works for example. This process is partially automated, the nodes include a basic program (default settings that assign them a valid Node ID) and default PAN IDs are defined specifically for this purpose in the concentrator. When this feature is selected, the network starts searching for nodes (which require manual activation) connected to that network, that once detected are configured and can be placed in its corresponding lamp. Therefore, the proposed solution demonstrates to be easily deployed and self-configured instead of a chain-based approach as this used in [5] where packet collisions are reduced at the cost of significantly complicating the regeneration of the chain when they existing nodes fail or new nodes are added.

### 5.4. Nodes and Regulation

Each end node of the system is formed by a commercial device, named a Waspmote, physically located inside the luminaries. Waspmote is a sensor board based on an ATmega1281 microcontroller that works at a frequency of 8 MHz and can operate with different wireless communication protocols. In our particular application, it provides the IEEE 802.15.4 RF transceiver for the 2.4 GHz unlicensed ISM band.

**Figure 9.**
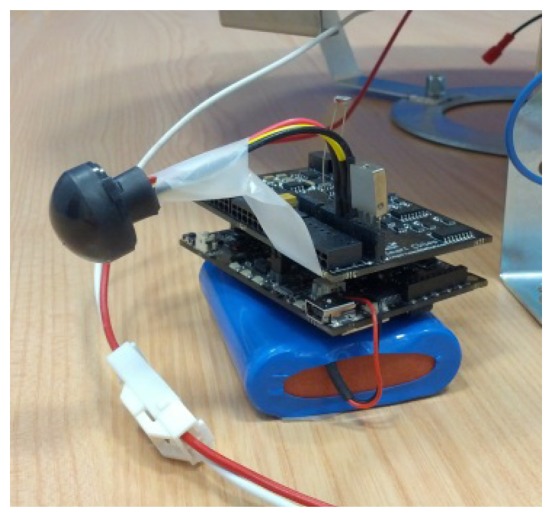
Picture of a full equipped node.

We distinguish two different end nodes, depending on the number of sensors they have connected: the full equipped nodes (see Figure 9) and the common nodes. The first ones have several sensors in order to capture and notify the instant environmental conditions in the surroundings of the nodes, in particular, a relative humidity sensor (808H5V5, Sencera Co. with an accuracy of ±4%), a temperature sensor (MCP9700A, Microchip, with an accuracy of ±2 °C) and a LDR type light sensor with a spectral range between 400 and 700 nm.

All the nodes include a passive infrared sensor (EKMC model from Panasonic) which can detect a presence at a maximum distance of 12 meters. The orientation of this sensor is a key factor to consider. In this particular scenario, the street only allows pedestrian flow on one side of the road, where the streetlights (and therefore the PIR sensors) are all along this sidewalk, so we have considered that people should walk from one end to the other, but might also cross the road and enter the sidewalk from any intermediate point. The proposed solution orients the first and last PIR sensors perpendicular to the sidewalk direction, in order to detect pedestrians as far as possible (10–12 m), and points the others below the luminary and slightly tilted towards the road, trying to detect both sides of the luminary and people at shorter distances (6 to 8 m).As an extra feature, there are some other critical signals monitored in the nodes. Each embedded system monitors both the luminary's current input and the output of the programmable driver, in order to know the instantaneous consumption of each spotlight and prevent malfunctions. Also, particular node's consumption and some XBee transponder′s inputs/outputs are monitored in order to promptly determine communication failures. A programmable Xitanium 150 W 0.35–0.7 A voltage source from Philips [34], provides power to each luminary, and allows the regulation of the light intensity in eight different levels. This regulation is performed by the Waspmote devices based on the following factors:

- Preset schedule, which will vary depending on the season and predetermined policies
- Weather conditions, obtained from the sensors measurements
- Detection of pedestrians in a node or in adjacent nodes

To minimize the data communication with the concentrator, each individual node stores the parameters which control its operation. These parameters (operating policies) contain information such as allowed lighting levels in different modes, communication parameters, conditions for generating events and expected behavior due to detection of pedestrian flow. These operating policies are designed and programmed to carry out this regulation depending on the area of action. This particular scenario represents one of the simplest ones. The whole system behaves in the following manner: during daytime, when the streetlights are powered off, the system devices are in sleep mode and only wake up periodically for monitoring activities. The sleeping period varies according to the season and it′s a setting that can be fixed remotely as well as by the section concentrator. When outdoor lighting facilities are turned on, the nodes are awake, with their sensors active, measuring and regulating the lighting depending to the preset policies, which configures different illumination levels considering that there is no need of public lighting on (at maximum power) when the streets are empty. In the valley hours, those which are intended to have minimum lighting, they are configured to a minimum level (level 1 or 2 of illumination, about 10%–20% of the maximum output of the driver). Both the minimum and maximum level (Level 7 or 8) of lighting intensities, are parameters which are set up according to the weather conditions obtained by the full equipped nodes (5th node in our particular scenario) autonomously or remotely. When one node detects a pedestrian on the sidewalk (detected by the PIR sensor) its lighting level increases and it informs the remote concentrator of pedestrian flow. If another detection occurs in a second node (a short period of time after the first one), this luminary will also start to increase its lighting level. After two positive detections, the concentrator will order the rest of the nodes of the current facility to rise their output, even if they have not yet detected the pedestrian flow. This increase of the emitting level is performed gradually, in order to facilitate eye adaptation to changing light conditions with the aim of avoiding unintended consequences such as glare that can affect both drivers and pedestrians, and that can occur if the transitions between different levels are performed too quickly or in inadequate stages.

### 5.5. Control Panel and Services

The control panel provides a Rich Internet Application (RIA) to control the operation of the sections of luminaries included in the system, generate activity reports and detect anomalies in the operation of any of the components in the system. This tool will be used by the municipal staff and has been developed taking into account two main features: (1) provide an easy to use friendly interface unlike conventional HMI/SCADA systems, (2) offer an accessible tool that allows system management from the municipal facilities, but also control the luminaries from mobile devices by maintenance personnel.

**Figure 10.**
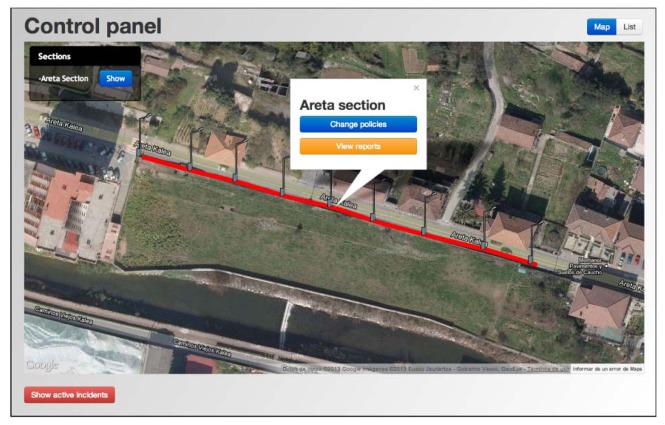
Web interface of the control panel.

The control panel has been implemented making use of different web development technologies: JavaScript, CSS3, HTML5, Ajax and jQuery, along with the use of the tools offered by Google for displaying and processing of geographic and positioning information and the Bootstrap front-end framework to facilitate the design of a responsive web that provides an optimal view across multiple devices and resolutions, as shown in Figure 10.

**Figure 11.**
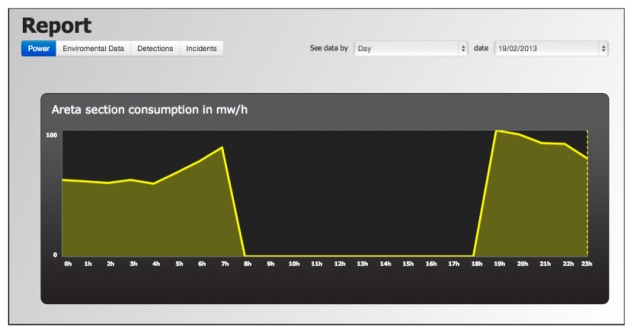
Consumption report in the analysis section.

The control panel offers three main features:

- *Incident Manager:* Allows authorized personnel to monitor the incidents automatically detected by the system due to anomalous consumption or loss of communication with nodes or sections of luminaries. This module responds not only to malfunctions, but it also conducts predictive maintenance based on the characteristics of each luminary built into the system. Thus, the maintenance service can provide for the acquisition of new lights that will need to be replaced at short notice.
- *Operating Policies Manager:* The control panel allows moditication of the main operating parameters assigned to sections of luminaries from simple forms. Users with rights to modify operation policies can select a set of lights individually or by sections, fill new values and request a change of the desired parameters. The changes are carried out asynchronously. When users request a change, they must wait for the concentrators that control some of the luminaries involved to request new policy changes and execute orders toward their respective luminaries. All concentrators consult periodically for policy change orders. The query latency can be programmed individually for each concentrator.
- *Reporting System:* This system provides the information collected in reports as depicted in Figure 11. Users can select a date range and a display area and the system will generate a detailed report showing the environmental conditions, consumption caused by street lighting and the activity detected. These reports can provide important information to assist municipal authorities in planning services and generate a reliable consumption audit.

### 5.6. Remote Control Center

**Figure 12.**
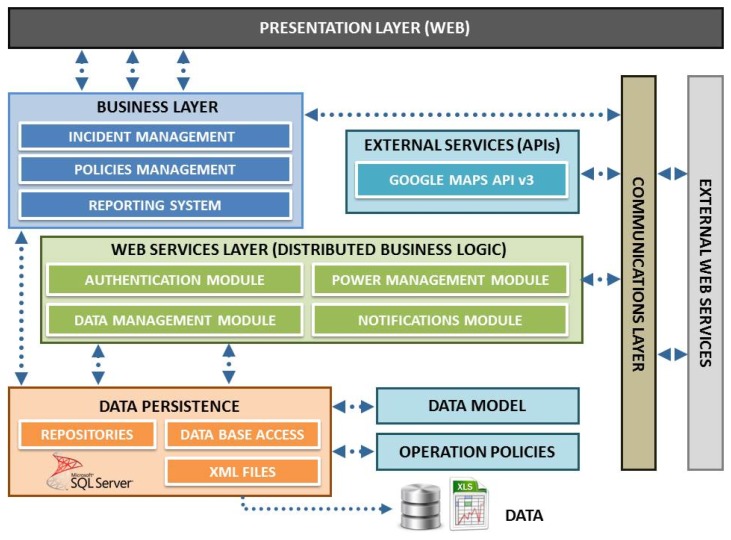
Architecture of the remote control system.

This project requires the development of a powerful back-end system whose architecture, which has been successfully tested in other smart environments [40], and can be visualized in Figure 12. Besides the web presentation and business layers treated in the previous point, there are other functional layers detailed below.

- *Web Services layer:* Developed by .NET technology, using WCF RIA Services, this functional layer is responsible for coordinating application logic between the middle tier and the presentation tier needed to meet the previously stated functional requirements. In addition it manages and gives the necessary permissions for the users of the system. The election of asynchronous web services for the distributed computing architecture facilitates scalability and simplifies the processes of the system improving reliability. However, it prevents real-time communication between the Remote Central Server and nodes.
- *Persistence data layer:* Within this layer all information transferred between the different elements of the system was modelled. Microsoft SQL Server 2008 DBMS has been used in this context. All remote concentrators deployed within our system are responsible for periodically send all information collected by successive sensors deployed in luminaries. XML files are also exchanged periodically in which concentrators report incidents detected and receive demanded changes in operating policies. Historical storage of this information will be used by the business layer.
- *Communication layer:* Information relevant to the application is in the database; however, access to such information is done through web services developed with Windows Communication Foundation (WCF) technology. This technical decision allows both data and application logic to be accessed from other devices, thereby ensuring the scalability and interoperability of the whole system. Security also is increased because the data access does not occur directly but through the services, providing greater control over database queries.

The network will be controlled at all times behind a firewall that prevents unauthorized access. The set of services developed allows full interoperability between the different components of the system, which is a major benefit in expanding the number of devices compatible with it and enabling their development in the future.

## 6. Test Results

In order to verify the viability and performance of the system, field tests have been performed in the selected scenario of Llodio. Two different kinds of tests have been carried out: communication tests between the various elements of the system and environmental data collection. Both are detailed in the following sections.

### 6.1. Communication Tests

The communications tests in the selected scenario were developed to guarantee the correct establishment of the channel link between all the deployed nodes in that section. Two opposite trials were tested. First the nodes were deployed forming a chain-topology network, measuring the signal quality between contiguous nodes. Node #1 is the initiator of the chain and sends a message to node #2 each 100 milliseconds. The message received at node #2 is retransmitted to node #3 and so on until node #7, which transmits it to the concentrator. Each node measures and sends in each message the received signal strength indication (RSSI) and the voltage of its battery, besides the information collected by its sensors. Tables 3, 4 and 5 summarize the packet error rate obtained for each node, the RSSI and number of messages lost by each node of the WSN, respectively. Results correspond to 20,000 messages sent by Node #1.

As it can be seen, two nodes exhibit a special behavior: #1 node and the concentrator node. On the one hand, the initiator node never loses a message, contrary to the gateway that suffers a small congestion while redirecting messages from the IEEE 802.15.4 to the IEEE 802.11 interface. However, the packet error rate is very low (0.13% for the worst case) and the distribution of losses by node is quite uniform.

Due to the morphology of the selected scenario, as opposed nodes of the full facility are at a distance within the coverage of the technology used (2.4 GHz), the second trial consisted in the deployment of a fully connected network. Node #1, placed at the first lamp of the section, sends messages directly to all the other nodes in the section (#2 to #7) with the same latency as in the previous test.

As it can be seen in Table 6, even if the received signal strength indication (RSSI) between the most distant nodes is within the sensitivity limit of the receivers (−100 dB), it is too close to the threshold, and the error rate is not acceptable (Table 7). Moreover, the streetlight section in which the system has been deployed has been recently installed and a future expansion is planned.

Given the results of the two tests performed, for the final deployment of the system a mesh network topology was chosen ensuring scalability and solving the cumulative error detected in the chain network. Tables 8 and 9 show the received signal strength indication and error rate in this approach.

### 6.2. Data Collection

Data collection was performed allocating several full equipped nodes in the luminaries of the section in a period of several weeks, with the unique purpose of obtaining data, not controlling the streetlights behavior for the moment. High-capacity batteries were used to power the prototype and the collected information was stored on memory cards.

Data from the ambient sensors was stored with a frequency of 5 minutes, every day from 17:00 pm to 09:00 am, a time period long enough to include the time that the lights are on in winter (from about 18:00 to 8:00). This data were verified later with nearby weather stations in order to test out their accuracy. During the same time, the pedestrian detection sensor was running and recording instantly the time when someone was detected. The detailed analysis of this data has been used to establish several important parameters in the system such as the minimum and maximum levels that will define the regulation of the luminaries, or the duration of the time range of these levels. They have also been used to specify the regular values of temperature, relative humidity and ambient brightness, allowing configuring limit values for the environment parameters. If one or more of these thresholds are exceeded, the system will launch alarms indicating that an abnormal situation has been detected, which can cause a change in the actual policies. Note that this analysis was performed in a particular season of the year (winter) and it would be necessary to extend it to a period of at least one full year in order to have enough data to improve the uninterrupted operation of the system.

**Figure 13.**
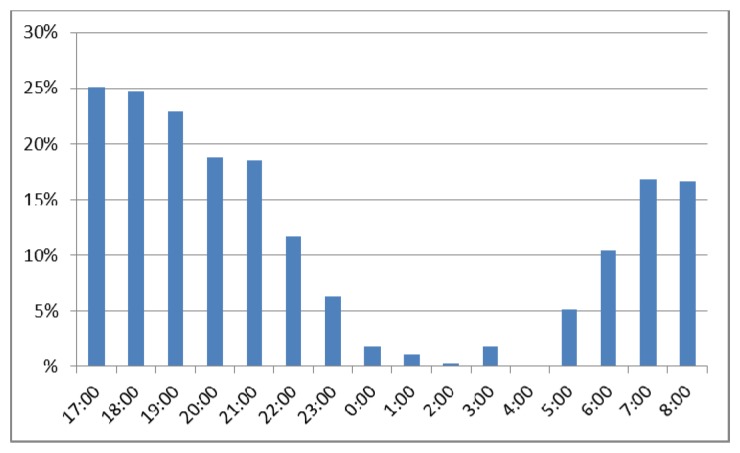
Distribution of pedestrian flow per day.

The chart below (Figure 13) shows the variation of the pedestrian detections per day relative to the minimum flow, which is detected during the late-night hours. As shown on Figure 13 the obtained variations per hour are a factor that varies greatly according the time slot. The highest flows of pedestrians are concentrated in the hours from twilight to 22:00 hours at night and between 7:00 to 8:00 on the morning, according to operation limits, whereas in off-peak hours very few people are detected, sometimes even none.

These noteworthy variations are expected to provide great energy savings when the system with our lighting regulation scheme is applied. Our strategy will consist of minimizing the lighting emissions in the off-peak hours, and therefore, the facility consumption, and only maintaining normal levels of lighting when needed (in terms of pedestrian flow) in order to accomplish the mandatory and legal requirements. Table 10 shows the consumption of a luminary according to its brightness level, accurately measured on-site. If no regulation is applied, the lights operate at the maximum level of luminosity, having a constant consumption during the time that the streetlights are on, even if there are no pedestrians walking through the sidewalk. This is the typical performance of the streetlight sections in our cities, but if regulation is applied, maintaining a minimum illumination (level 2) when there are no detections, and gradually increasing it to a maximum level (level 7, or 8) when a pedestrian is detected, an optimized consumption is guaranteed at any time of the night.

**Figure 14.**
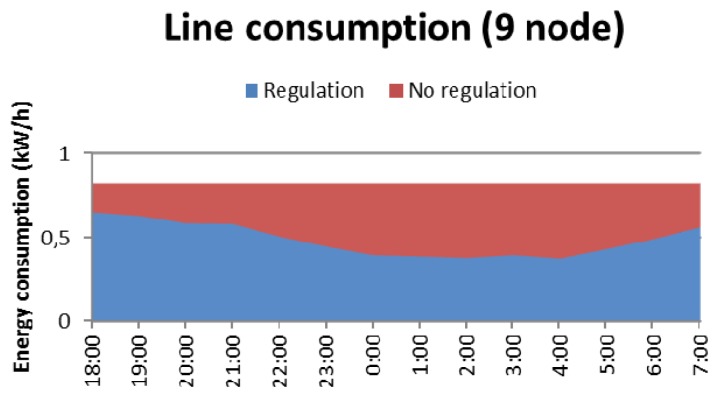
Energy consumption of the line (9 nodes).

The achieved energy savings can be checked by looking at the graph below (Figure 14). This graph shows the energy consumption of the whole line *versus* time, when applying the regulation (in blue) and without applying it (red). It is obtained calculating the mean detections shown in the graph above Figure 14 clearly shows that the consumption is always lower when the regulation is applied than if not. As expected, the decrease in energy consumption is higher in the time slots during which there are fewer pedestrians on the street.

In particular, the energy consumption of the entire line of nine lights for a week without applying any regulation is 98 kW/h and using the described regulation it is 67 kW/h, implying that the achieved average savings are around 40%. Note again that these savings will vary depending on the season because both luminosity and the number of pedestrians walking though the sidewalk vary with the time of the year.

The obtained experimentation values have been extrapolated to a whole year's performance (4.327 h of functioning, a little more than the 4.315 h recommended by the Ministry) and then, compared with the available data of the energy audit done by the Spanish company Consultoria Lumínica (http://www.consultoria-luminica.com) in 2010. According to this document, each spotlight consumes 171 W/h. Our tests, made at the Llodio scenario, reveal that the consumption per luminary is a little bit lower, as shown in Table 11. The presented values were obtained stating the instant current variations in samples of several hours, using oscilloscopes and current probes in order to measure the current feeding one luminary. The line consumption was also measured (in the electric cabinet) and the section values were lower than the ones presented by the previously made energy audit.

The energy audit data conclude that the entire section is efficient (it has been rated as type A, within a standardized ranking list where A is the best and G the worst one in terms of efficiency), with an annual consumption near 6,660 kWh. In fact, the existing devices, that include the constant current driver in order to feed each luminary, barely consume 132 W/h, so the annual consumption is lower, as shown in Table 12, second row, “Without Regulation (measured data)”. If we compare these values with the ones we expect to achieve using our system for one whole year, the results in terms of reduction of energy consumption and tons of CO_2_ per year are quite inspiring, as stated in Table 12.

As previously mentioned, the savings could vary between the 30%–40%, according to the real performance of the system during a whole year.

## 7. System Extensions in Progress

Once the streetlight system has been developed and preliminary test results in a real scenario have been obtained, some extensions of the system are being explored in order to improve it. In this section we will describe the work in progress towards this target. The first extension is a fuzzy inference system integrated into the sensor nodes which is able to make better decisions and improve the fault tolerance and scalability, due to the fact it would not require any supervision of a remote control system. The second one is a customized fully-integrated antenna which is being designed to maximize space, reduce costs and possible visual impact of antennas in street lamp-posts.

### 7.1. Autonomous Adaptive Embedded System based on Fuzzy Logic

**Figure 15.**
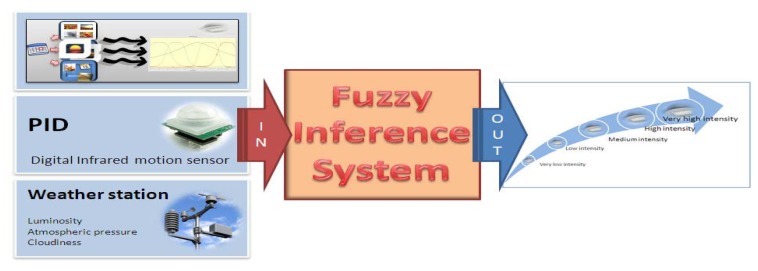
Information fusion by the embedded fuzzy interference system.

The information harvested by the set of wireless sensors devices allows the detection and reporting of incidents in real or delayed time (depending on requirements) and is used to decide the operation mode of the lamp, but it has allowed us to build a knowledge base that allows intelligent decision making by means of an expert system, using approaches such as the one presented in [41]. The main challenge of this first proposed extension of the system is to provide better autonomous response to a changing environment and situations without requiring any supervision of a higher order entity. Sometimes environmental information is inaccurate since the sunlight, the season and the UV index, among others, may affect the measurements considerably, and therefore the decisions. Therefore, it comes to getting the most complete and accurate information as possible and trying to supplement that information with that obtained by other sensors in order to minimize possible noise and achieve adequate situational awareness. For such reasons we are exploring an improvement of the system which lies in a fuzzy system in charge of information fusion used for supporting decision making which cooperates with an ontology charged in the sensor as depicted in Figure 15.

**Figure 16.**
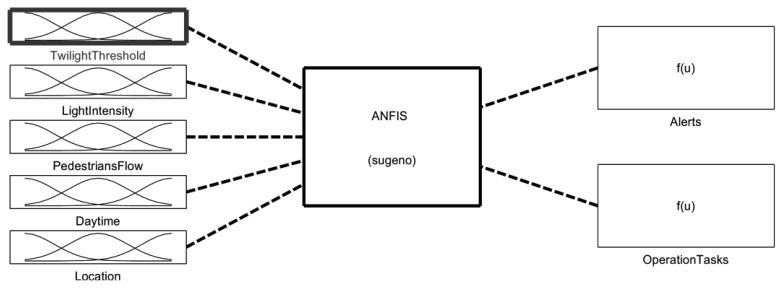
Adaptive neuro-fuzzy inference system (Sugeno).

The lighting system managers can set up a number of rules concerning the operation, certain thresholds, some power consumption limits, *etc.*, but they could also seek advice from an expert system that learns continuously through the knowledge stored automatically and the operation information stored in the system. With the aid of an adaptive neuro-fuzzy inference system (ANFIS) we can obtain a set of rules that can be easily integrated in the sensor node since the computational cost associated to its utilization is really low. We built a Sugeno-type fuzzy inference system with the aid of Matlab and its fuzzy logic toolbox. The ANFIS, depicted in Figure 16, is trained with a large set of measurements and the neural network provides as output a set of fuzzy rules that can be embedded in the sensor.

**Figure 17.**
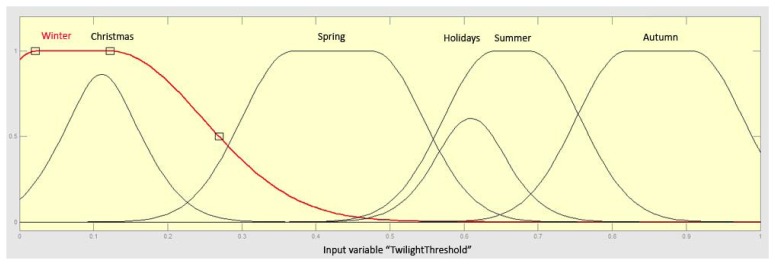
Fuzzy sets referring the twilight threshold (first input variable of the ANFIS).

The remote control system may deal with large data flows and may process complex rules involving large data sets, something that happens when using statistical information for planning new operating guidelines, but that it is impossible to carry out with the limited computing and storage capacities of a sensor. Although many other input parameters may be considered, our proposal considers five: twilight threshold, light intensity, pedestrian flow, daytime and location. The twilight threshold input refers to the light due to the illumination produced by the sun when it is slightly below the horizon. In our case, we consider the civil twilight. Figure 17 shows the fuzzy sets corresponding to this last input (in red), where four sets concern the seasons of a year and two special periods like Christmas and holidays (*i.e.*, local festivities) when the street lighting is greater. The rest of inputs perform in the same way. Information concerning pedestrian flow is provided by de digital infrared motion sensor (PIR) located in the mote. Daytime is also provided by the clock located in the mote. The light intensity information could be provided by a remote weather station through a wireless communication channel. And location information could be stored in the mote during its deployment, or even a GPS transceiver can be connected to the mote if the device mobility justifies this additional cost. The ANFIS considers two output variables: alerts and operation tasks. The first one is a constant, which is activated whenever a dysfunction or abnormal environmental situation is detected. The second one provides eight constant output values indicating the graduation in the lightning that must be performed (from 1 to 8). The selection of a Sugeno-type ANFIS is due to Sugeno's either linear or constant output membership functions. In this case, we have selected as “*And method*” the product and as “*Or method*” the operator “*probor*” for each one of the four inputs. The method selected for the defuzzification is “*wtaver*” and the “*maximum*” is the method used for the aggregation. The initial model for ANFIS training is created by first applying subtractive clustering on the data, and the ANFIS is trained using the hybrid optimization method (a combination of least-squares and back-propagation gradient descent method) provided by the fuzzy logic toolbox of Matlab.

**Figure 18.**
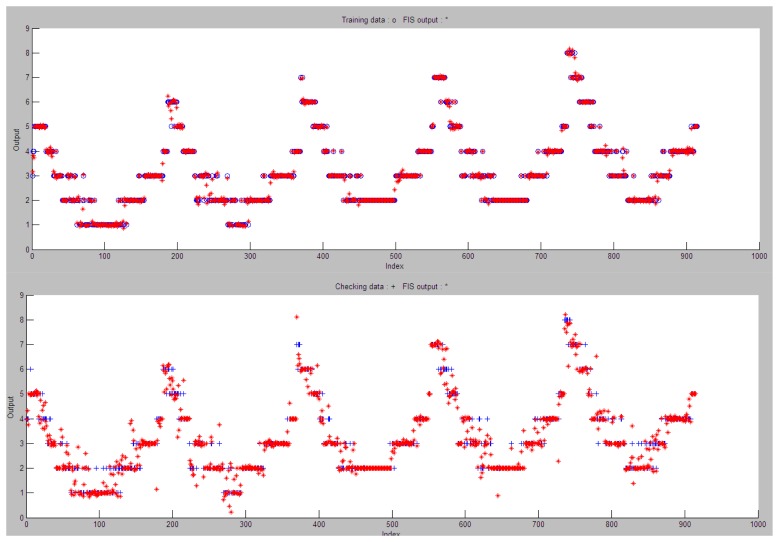
Evaluation of the ANFIS for the training (up) and testing (down) sets.

Figure 18 shows the results obtained for the selected ANFIS when validating the system with the training and testing sets. Obviously, good classification values are obtained for the training set of samples, but so are those obtained for the testing set. Both training and testing sets have been obtained according to the sunrise and sunset, luminosity and twilight values obtained for the city of Pamplona (Spain) on the year 2012 according to the open data provided by the Gobierno de Navarra (meteo.navarra.es) and the Agencia Estatal de Meteorología (www.aemet.es).

**Figure 19.**
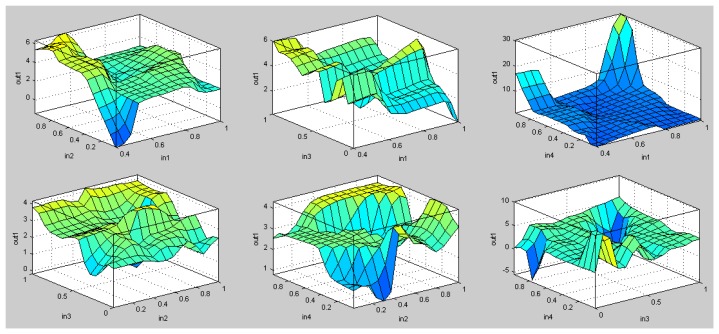
Surface comparison between the input values of the ANFIS.

The total amount of samples obtained refers to the 366 days of the year 2012, where different values of location and pedestrian flow are considered. Both training and testing sets have the same number of samples and follow a uniform distribution. Figure 19 shows the surface comparison between the input values of the ANFIS obtained.

**Figure 20.**
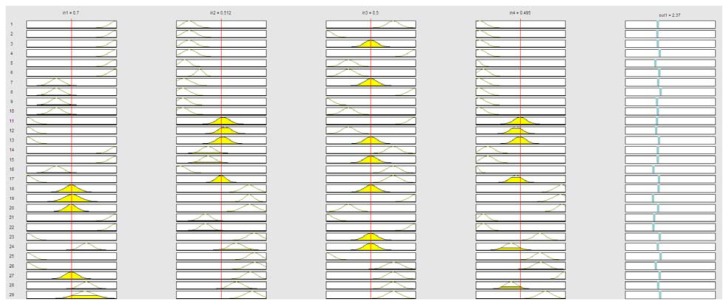
Rules provided by the ANFIS for *operation tasks* output.

Since the proposed ANFIS provides as outputs a set of IF-THEN rules, the computational cost of a distributed approach is not high enough, and the expert system can be embedded into the mote in order to drive the lamp. As illustrated in Figure 20, we obtain a set of rules related to the operation tasks to be performed to manage the intensity of the lamps. Note that each node applies these rules to manage the lamps it has in charge. Figure 20 shows the graphical interface provided by Matlab to visualize the rules, but these rules are embedded in the nodes in terms of IF-THEN sentences, for example: *If (in1 is in1cluster7) and (in2 is in2cluster7) and (in3 is in3cluster7) and (in4 is in4cluster7) then (Alerts is alertcluster1) and (OperationTasks is operationtaskscluster7)*.

According to the operation scheme of the system three different approaches may be considered. A centralized operation consists of the location of the decision logic (both expert decision and management/monitoring systems) on a unique, well communicated site where all the information is collected, achieved and accessed in a centralized way. This approach centralizes information and decision, and makes final nodes act as unintelligent devices merely following orders simplifying the hardware and software necessary. A distributed scenario focuses on the autonomous decision at the motes according to some parameters that may be configured (and monitored) from a centralized management system. In this case, the complexity of the motes increases, but allows a better response to sudden and local changes, and a significant decrease on the number of messages transmitted (leading to energy savings). It also allows a greater fault tolerance than the one provided by the centralized approach where the management system is the bottleneck and a possible point of failure. Of course, a hybrid approach can also be considered in order to maximize the benefits of the previous approaches. The system can perform in a centralized way in regular operation switching to a distributed behavior when a fault of the communications or the management system occurs.

Since the proposed ANFIS provides as outputs a set of IF-THEN rules, the computational cost of a distributed approach is too high, and the expert system can be embedded into the mote. The knowledge base may be continuously increased by monitoring the environmental scenario, the measure of the degree of satisfaction of users (surveys) and the assessment of the energy consumption. The operation knowledge may be achieved and reevaluated on demand if needed and the set of new rules updated into the motes through the wireless communication network.

### 7.2. Fully-Integrated Antenna Design

To maximise space, reduce costs and the possible visual impact of antennas in street lamp-posts, and contribute towards less-bulky camouflaged units, an antenna is being customised to satisfy the demands of the planned streetlight system. Hidden prototypes are very attractive as they are considered environmentally friendly [42]. In addition, interest is shown to re-utilise the design to harvest energy from ambient electromagnetism specifically due to the power savings implications involved [43]. Generally, lamp-posts are self-electrically supported, but where solar-panels are used, the rectenna can contribute positively to battery savings and self-powering the incorporated Zigbee units. The energy can be collected by intentionally exploiting the reception of nearby bands to that of the antenna resonating frequency of interest. To meet the demands immediately outlined, a self-integrated for low-cost and camouflage antenna-rectenna is being developed. For the realisation, the chassis of the lamp-post is re-used as the main electrical conductor. This is, an antenna is fully-integrated in a commercially available lamp-post, from Simon Lighting [35], by effectively re-utilizing the actual steel made case, shown in Figure 18. Complex engineering practices (including additional conductor/compounds) are high costly and unwelcome, e.g., unlike the proposed design, a traditional antenna requires drilling the lamp-shade for the feed.

The antenna is being customized for use in Zigbee broadcasting and networking applications and using the (worldwide) unlicensed 2.4 GHz band of the IEEE 802.15.4 standard. The prospective camouflaged lamp-post antenna is subsequently detailed. Previously, the adherence of copper particles [42] to a metallic chassis to form the electrical conductors of antennas has been used, but due to the close proximity to the metal plane, they are inefficient in this application.

**Figure 21.**
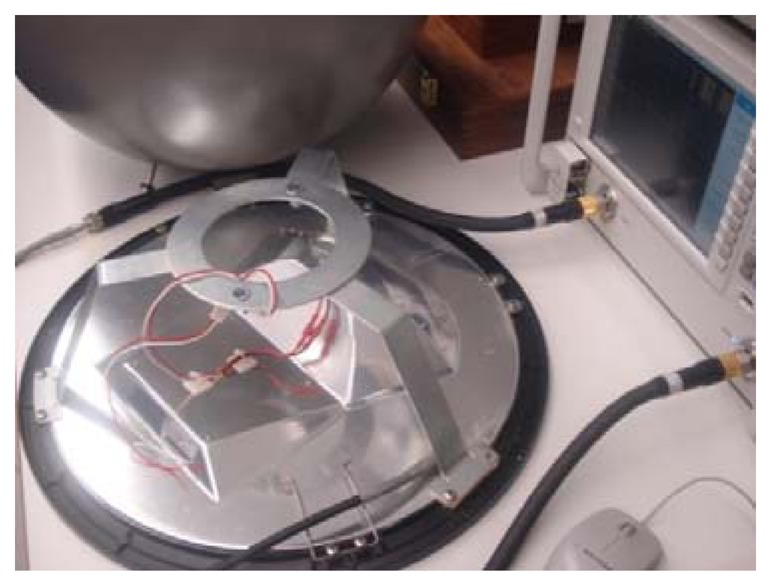
The lamp-post as an antenna showing the radiating elements seen from inside the lamp.

A snapshot of the considered lamp-post Planar Inverted-F Antenna (PIFA) is shown in Figure 21, depicting the metal chassis and internal elements for the realisation of the advanced “economical” solution. The prototype, whose total dimensions are dictated by the lamp-post geometry, needs no size extension. The principal (already existing) conducting elements inside the cavity of the lamp-post are given in the figure and an additional screwable string is used for fine-tuning the antennas′ impedance matching. The feed is located inside the lamp-post (Figure 21) to provide easy interface connectivity to any potential front-end hardware placed inside the lamp-post enclosure; a flexible coaxial cable terminated by an SMA connector is used for the transition.

**Figure 22.**
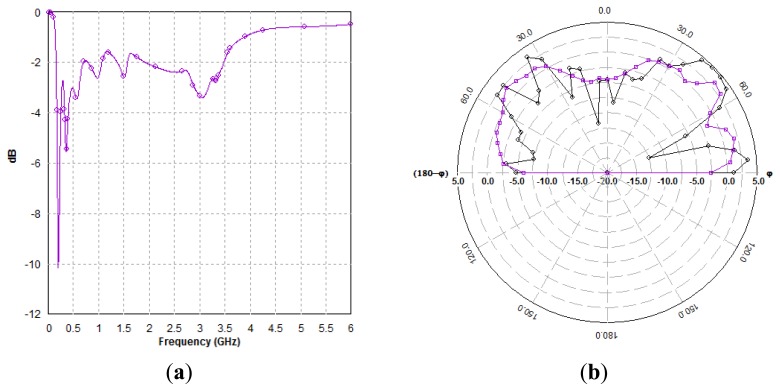
Simulated RL (**a**), and radiation patterns, E-Total (**b**) of the antenna.

The structure was modelled and simulated using Zeland IE3D. Preliminary simulated results are now presented. It can be established that an initial antenna design operating in the 2.4 GHz band having a total fractional bandwidth is sufficient for the application, however, re-tuning is needed to improve the impedance matching of the antenna. Figure 22(a) shows the return loss (RL) of the antenna with a response of about −2.2 dB for the upper and lower frequencies–yet the shortcoming, needs to be amended by tuning.

Preliminary radiation patterns in polar form for the azimuth and elevation planes at 2.4 GHz are presented in Figure 22(b). The pattern is truncated at the rear direction 180° due to the lamp-shade being used as a large reflector. The response is attractive for the application that needs to be seen by neighbouring lamp-posts communicating peer-to-peer and likely mobile equipment in the vicinity. Results predict 5 dBi gains at −2.2 dB RL and peak gains of 17 dBi are possible at lower beamwidths. Concluding remarks indicate that the proposed antenna has the potential to be further tuned so that the load of the antenna is properly matched and therefore be adequate for this setting where the lamp-post itself is utilised as a radiator and reciprocally as an energy harvester.

## 8. Conclusions

An intelligent street lighting system with high degree of adaptability and ease of installation is presented in this paper. The system takes advantage of a centralized control strategy and the use of wireless connection elements to achieve increased energy efficiency as well as viable communication links in complex scenarios.

One of the main challenges in such a street lighting system is the influence of the intrinsic characteristics of each installation (size, morphology, lamp technology, *etc.*) which require for the proper deployment of such a system an individualized study of the electromagnetic spectrum and the behavior of the wireless channel in order to optimize the performance of the communication links.

The proposed system can accommodate to many different realities of each municipality ensuring scalability, interoperability and accessibility (in the sense that the system is accessible from multiple platforms: mobile phone, PC, tablet), and its ease of deployment, with the case study of its implementation in a section of street lights in the town of Llodio in Northern Spain.

At the present stage, the system has been completely deployed and is being validated in the final scenario. During the next months, acquired data will be analyzed in order to establish optimal power generation policies and optimize the learning rules of the fuzzy system. The implementation of an autonomous system and the possibility of integration of customized antenna in the surface of the luminary, are expected to be included in the final system implementation with great prospects for success.

## Figures and Tables

**Table 1.** Technical specs of the ALYA LED.

Parameters of The Luminary			
IP/IK Ratio	IP 66/IK 09	Colour Temp.	5,500 K
Class	Class I/Class II	Flux	4,700 lm
System	350/530 mA	Efficiency	87,43 lm/W
Voltage	230V AC	CRI	>75 Ra
W	54	ULOR	E1(<1%)
Frequency	50/60 Hz	Working Temp.	−20 °C to 50 °C
Leds number.	48 LEDS	Lifespan	50.000 h (L70B10)

**Table 2.** Simulation parameters.

**Parameters in the Ray Launching Simulation**	
Frequency	2.4 GHz
Cuboids resolution	20 cm
Vertical plane angle resolution Δ*θ*	1°
Horizontal plane angle resolution	1°
Reflections	5
Transmitter Power	100 mW

**Table 3.** Packet error rate (PER) by node.

**PER by Node**						
#1	#2	#3	#4	#5	#6	#7
0.000%	0.130%	0.065%	0.080%	0.130%	0.120%	0.120%

**Table 4.** Received signal strength indication (RSSI) for each node performing the 1st test.

**RSSI** **(dBm)**	**Nodes**						

	**#1**	**#2**	**#3**	**#4**	**#5**	**#6**	**#7**
**Average**	0.0000	−58.0382	−61.5182	−60.0677	−59.8993	−62,3874	−62.3792
**Std. Deviation**	0.0000	0.4750	0.6043	0.4655	0.5829	0.5270	0.5189

**Table 5.** Number of messages lost by each node of the chain topology WSN.

**Messages lost**	**Nodes**						

	**#1**	**#2**	**#3**	**#4**	**#5**	**#6**	**#7**
Average	0.0000	0.0013	0.0020	0.0028	0.0041	0.0053	0.0065
Std. Deviation	0.0000	0.0376	0.0466	0.0563	0.0669	0.0786	0.0859

**Table 6.** Received signal strength indication (RSSI) during the 2nd test.

**RSSI** **(dBm)**	**Nodes**						

	**#1**	**#2**	**#3**	**#4**	**#5**	**#6**	**#7**
**Average**	0	−57.248	−72.945	−75.068	−93.554	−95,3874	−98.846
**Std. Deviation**	0.0000	0.5250	0.4775	0.5650	0.6025	0.5450	0.6675

**Table 7.** Number of messages lost by each node of the fully connected topology WSN.

**Messages lost**	**Nodes**						

	**#1**	**#2**	**#3**	**#4**	**#5**	**#6**	**#7**
**Average**	0	0.001	0.003	0.0038	0.0452	0.0440	0.058
**Std. Deviation**	0.0000	0.0375	0.0425	0.0475	0.0550	0.0650	0.0875

**Table 8.** Received signal strength indication (RSSI) using the DigiMesh protocol.

**RSSI** **(dBm)**	**Nodes**						

	**#1**	**#2**	**#3**	**#4**	**#5**	**#6**	**#7**
**Average**	0	−58.460	−66.850	−67.035	−66.948	−66,876	−67.487

**Table 9.** Number of messages lost by each node in the DigiMesh WSN.

**Messages Lost**	**Nodes**						

	**#1**	**#2**	**#3**	**#4**	**#5**	**#6**	**#7**
**Average**	0	0.001	0.002	0.002	0.002	0.002	0.002

**Table 10.** Consumption of one luminary.

**Light Level**	**Driver Consumption (mA)**	**Energy Consumption (kA/h)**
1	144	3,31E–02
2	172	3,96E–02
3	206	4,74E–02
4	244	5,61E–02
5	286	6,58E–02
6	328	7,54E–02
7	364	8,37E–02
8	396	9,11E–02

**Table 11.** Energy consumption.

**Energy Consumption**	**Per Luminary**	**Per Line**
Without Regulation (Audit data)	171 Wh	1,539 kWh
Without Regulation (Measured data)	132 Wh	1,188 KWh
Applying Regulation (Extrapolation)	90.9 Wh	818 Wh

**Table 12.** Annual comparison.

**Energy Consumption**	**Energy Consumption CO_2_**	**Energy Consumption**
Without Regulation (Audit data)	2.6 Tons of CO_2_	6,659 kW
Without Regulation (Measured data)	2.0 Tons of CO_2_	5,143 kW
Applying Regulation (Extrapolation)	1.38 Tons of CO_2_	3,540 kW
